# Toxicity to the Male Reproductive System after Exposure to Polystyrene Nanoplastics: A Macrogenomic and Metabolomic Analysis

**DOI:** 10.3390/toxics12080531

**Published:** 2024-07-23

**Authors:** Xue Zhang, Yueping Wu, Xufeng Fu, Shulan He, Liping Shi, Haiming Xu, Xiaojuan Shi, Yue Yang, Yongbin Zhu, Yanrong Wang, Hongyan Qiu, Hongmei Li, Jiangping Li

**Affiliations:** 1 Department of Epidemiology and Health Statistics, School of Public Health, Ningxia Medical University, Yinchuan 750004, China; 18706911052@163.com (X.Z.); wuyueping202206@163.com (Y.W.); heshulan0954@163.com (S.H.); slp970408@163.com (L.S.); 2021031045@nxmu.edu.cn (X.S.); yangyue2705@163.com (Y.Y.); 18509552164@163.com (Y.Z.); wyrong0609@163.com (Y.W.); yanzide80@163.com (H.Q.); 2 Key Laboratory of Fertility Preservation and Maintenance of Ministry of Education, School of Basic Medical Sciences, Ningxia Medical University, Yinchuan 750004, China; fuxufeng100@163.com (X.F.); 20160133@nxmu.edu.cn (H.L.); 3 Department of Occupational and Environmental Hygiene, School of Public Health, Ningxia Medical University, Yinchuan 750004, China; 20150143@nxmu.edu.cn; 4 Key Laboratory of Environmental Factors and Chronic Disease Control, Ningxia Medical University, Yinchuan 750004, China

**Keywords:** nanoplastic, reproductive, macrogenomic, metabolomic, gut microbiota

## Abstract

Nanoplastics (NPs) cause serious contamination of drinking water and potential damage to human health. This study aimed to investigate the effects of NPs with different particle sizes and concentrations on the reproductive function of male mice. In this study, free drinking water exposure was used to expose male BALB/C mice to PS-NPs (20 nm, 200 nm, and 1000 nm) at 0.1 mg/L, 1 mg/L, and 5 mg/L for 4 months. The male reproductive function of the mice was assessed after NPs exposure, and fecal and blood samples were collected for macrogenomics and metabolomics. The results showed that PS-NPs resulted in mice with reduced testicular organ coefficients, decreased sperm quality, altered testicular tissue structure, disturbed sex hormone levels, and abnormal levels of inflammatory factors and oxidative stress. Furthermore, this study found that NP exposure affected the alteration of gut communities and metabolic pathways related to male reproduction, such as Clostridium and glutathione metabolism. Importantly, we found an effect of NP particle size on reproductive function. In the future, more attention should be paid to the smaller particle sizes of NPs.

## 1. Introduction

Polystyrene (PS) is a common plastic material often made into household products that are extensively encountered by humans, such as disposable plastic cups, feeding bottles, and masks [1]. Disposed PS enters the environment and, after a long period of degradation, breaks down to form PS microplastics (PS-MPs) and nanoplastics (PS-NPs) [2,3]. Recently, a study found that 36% of the micro-nanoplastics (MNPs) present in the human body are from PS [4].

The aquatic environment has been hit hardest by the contamination of NPs, which often find their way into drinking water through various pathways [5]. In 2018, a team of investigators in the United States collected 159 tap water samples from 13 different countries, 81% of which were found to contain NPs [6]. In January 2024, a Columbia University study showed that three of the best-selling brands of plastic bottled water on the U.S. market contained approximately 110 to 370 thousand MNPs per liter of water, 10% of which were microplastics, and the remaining 90% of which were smaller, nanoscale plastics [7]. Some studies have shown that NPs found in bottled water are usually in the size range of less than 1 μm and may even be less than 100 nm [8]. Previously, these studies demonstrated biological health impairments caused by waterborne exposure to MPs, such as Jin’s study showing that drinking water exposure to MPs resulted in neurotoxicity in mice [9] and Chen’s study demonstrating that mothers exposed to MPs in drinking water had offspring with metabolic disorders [10]. Additionally, it has been demonstrated that waterborne exposures lead to more MP enrichment in organisms compared with foodborne exposures [11]. Furthermore, MNPs also have other additives and absorb a lot of other endocrine disruptors from the aquatic environment [12,13]. These chemicals along with MNPs synergistically increase the toxicity of MNPs [14]. In conclusion, the health hazards associated with exposure to drinking water contaminated with MPs deserve further study.

To the best of our knowledge, research at this stage has amply demonstrated that MNPs interfere with the normal reproductive functions of living organisms. Previous studies have shown that PS-MPs at 2 μm and 6 μm resulted in decreased sperm viability and oocyte counts in the oysters [15]. Jin’s study used 0.5 μm, 4 μm, and 10 μm PS-MPs, which resulted in impaired spermatogenesis, decreased testosterone levels, and inflammatory responses in the testicular tissues of male mice [16]. Xu’s study on mice used 25 nm, 50 nm, and 100 nm PS-NPs and similarly found reproductive dysfunction [17]. However, most studies at this stage have not been able to demonstrate systematically the existence of a size effect on the harm caused by NPs, especially in terms of reproductive health damage.

Although the majority of studies at this stage have demonstrated the harmful effects of NPs on male reproductive health, the exact mechanisms are still somewhat controversial. Some studies have shown an inextricable link between gut microbial homeostasis and male reproductive function [18,19]. Previous studies have suggested that androgens are involved in regulating the gut microbiome, which has been found to regulate primarily androgen production and metabolism. Moreover, oral exposure is one of the most important ways that NPs enter the body, and the gastrointestinal tract is considered the first organ to be exposed. Previous studies have found that oral exposure to NPs significantly alters the abundance level of the intestinal microbiota, inducing dysbiosis and related metabolic disorders in the gut microbiota [20]. In contrast, altered gut microbiota and metabolism by NPs may contribute to androgenic dysfunction by inducing immune activation and insulin resistance or by causing dysregulation of sex hormone levels and disruption of the testicular microbiota [21].

This study used 20 nm, 200 nm, and 1000 nm PS-NPs as the primary study materials and set three dose concentrations based on previous studies (0.1 mg/L, 1 mg/L, 5 mg/L) [22]. Free drinking water exposure was used to simulate health damage systematically from realistic exposure to PS-NPs. This study explored male reproductive damage mediated by gut microbiota and proved particle size effects in the hazards of NPs. The results provide new insights into the future assessment of the potential health risks of PS-NPs to human health and a theoretical basis for preventing human reproductive health damage.

## 2. Materials and Methods

### 2.1. Materials

PS-NPs of 20 nm, 200 nm, and 1000 nm with uniform particle size and monodisperse distribution were used as the primary materials for this study. The PS-NPs were purchased from Yuan Biotech (Yuan Biotech, Shanghai, China) and were prepared by adding pure water to the desired concentration for free drinking exposure to mice.

### 2.2. Exposure and Animals

**Figure 1:**
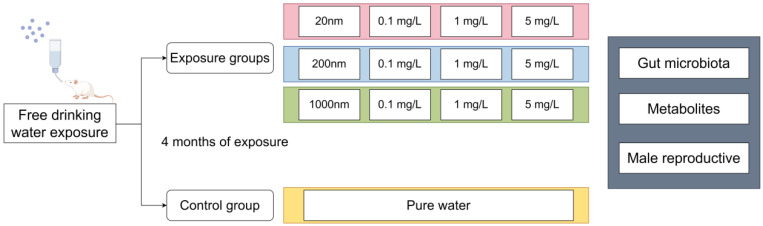
Study subgroup design. Note: The figure was drawn by Figdraw at https://www.figdraw.com/static/index.html (accessed on 31 December 2023).

The exposure was divided into nine groups using particle sizes and doses of PS-NPs; the control group used the same volume of pure water. The exposure groups were categorized according to the particle size and concentration of the intervening PS-NPs into 20 nm–0.1 mg/L, 20 nm–1 mg/L, 20 nm–5 mg/L, 200 nm–0.1 mg/L 200 nm–1 mg/L, 200 nm–5 mg/L, 1000 nm–0.1 mg/L, 1000 nm–1 mg/L, and 1000 nm–5 mg/L groups. All mice were used in a free drinking exposure mode without forced changes in their drinking habits. The specific exposure groups are shown in Figure 1.

Seventy male BALB/C mice (five weeks old) were used in the current study (Licence number: SCXK 2019-0008). The mice were purchased from the Animal Center of Ningxia Medical University and approved by the ethics committee (Ethical approval number: IACUC-NYLAC-2022-188). The initial body weight of mice was 16.3~19.8 g, and they were divided into ten groups of seven mice each using simple randomization. Seven mice were housed in the same cage and kept in the SPF Class Animal Center of Ningxia Medical University, maintained at 22 °C, 70% humidity, and fed under a 12:12 h light–dark cycle. All mice were provided with standard laboratory animal feed purchased from Jiangsu Synergy Bio (Nanjing, Jiangsu, China). After an initial adaptation to the cage, food, and laboratory conditions for one week, the mice were exposed to different concentrations and particle sizes of PS-NPs in drinking water for 4 months, during which time body weight was measured and recorded every 7 days. During the experiment, we renewed drinking water every night; after adding PS-NPs and mixing, each water bottle was ensured to be at 250 mL. The quantity of water consumed by each group of mice was monitored during the first month of exposure. All manipulations in animal experiments followed the 3R principles and minimized animal suffering.

### 2.3. Sample Collection

Fecal samples were collected from each mouse within 12 h using mouse metabolic cages, with no food or water supply, to analyze gut microbiology after four months of exposure. The collected fecal samples were placed in a separate sterile cryotube and stored in a −80 °C refrigerator before use. In this study, skimmed cotton wool was soaked in 5–10 mL of ether and used to line the bottom of a glass container. The animal was then placed inside, the lid was closed, and the animal entered the anesthesia state within 20–30 s. Blood samples were collected by removing the eyeballs. Epididymis and testis tissues were collected and weighed to calculate the organ coefficient from all mice (Organ coefficient = organ weight/total body weight). The blood that coagulated in the upper serum layer was collected after standing at room temperature for 30 min and stored in a −80 °C refrigerator for metabolites analysis.

### 2.4. Sperm Quality Observation

Under aseptic conditions, the epididymis was isolated in a culture dish containing normal saline, with exterior adipose tissue detachment, and rinsed well with normal saline to avoid interfering with the observation of sperm. Subsequently, the epididymis of each mouse was preserved in a centrifuge tube containing 1.0 mL of physiological saline and placed in a 37 °C incubator for 10 min. The sperm suspension was filtered into a 200-mesh sieve in 1.5 mL centrifuge tubes. Then, 10 μL of sperm suspension was taken with a pipette and dropped on a hemocytometer. The spermatozoa were rapidly observed under a microscope, and sperm were counted and analyzed for sperm viability using an animal semen analysis system (Licence number: 2102200762, SAS Medical, Beijing, China). Three fields of view were observed for each semen sample to assess the sperm count and viability of each mouse (head threshold: 260, body threshold: 700, scale: 1:205, density correction factor: 0.475).

The sperm deformity rate was calculated using the eosin staining method. First, 10 μL of sperm suspension was sucked up on a clean slide with a rubber-tipped burette and pushed uniformly, then the slide was dried and fixed with methanol for 5 min. After drying, the smear was stained with 1% eosin solution for 15 min, rinsed gently with tap water, and dried for microscopic examination. Two hundred sperm were counted randomly under a microscope, and the sperm deformity rate was calculated.

### 2.5. Analysis of Inflammatory Factors, Testosterone, Follicle-Stimulating Hormone, and Oxidative Stress

Testicular tissue from the mice was weighed accurately, a 10% homogenate was prepared under ice-water bath conditions, and the supernatant was taken for determination. The Mouse IL-6 ELISA Kit (Servicebio, Wuhan, Hubei, China) and the mouse TNF-α ELISA Kit (Servicebio, China) were used to determine the inflammation level in mouse testicular tissues. An enzyme-linked immunosorbent assay kit for testosterone (Uscn Life Science Inc., Nanjing, Jiangsu, China) was used to determine testosterone levels in mouse testicular tissues. An enzyme-linked immunosorbent assay kit for follicle-stimulating hormone (FSH) (Cloud-Clone, Wuhan, Hubei, China) was used to determine FSH levels in the mouse serum samples. Moreover, malondialdehyde (MDA), superoxide dismutase (SOD), glutathione (GSH), and catalase (CAT) (Servicebio, China) were used to detect oxidative stress in mouse oxidative stress levels in testicular tissues. The operation was carried out strictly according to the kit’s requirements (See Supplementary Materials S1.1 for further details).

### 2.6. Immunofluorescence Staining

Immunofluorescence was used to analyze changes in the expression and distribution of TNF-α and IL-6 in the testis. Testicular tissue was fixed in 4 um thickness sections using paraffin sections. All operations were performed following A Guide to Successful Immunofluorescence by Cell Signaling Technology, Inc. (Boston, MA, USA). The primary antibodies mouse anti-TNF-α (antibody dilution: 1:200) (Servicebio, Wuhan, China), mouse anti-IL-6 (antibody dilution: 1:2000) (Servicebio, Wuhan, China), rabbit anti-MCP-1 (Servicebio, Wuhan, Hubei, China), and rabbit anti-cXCL10 (Servicebio, Wuhan, China) were used. The primary antibody was incubated overnight at 4 °C. PBS was washed 3 times and then exposed to a secondary antibody. Secondary antibodies HRP goat anti-rabbit and CY3 goat anti-rabbit were used in this study. The secondary antibody was incubated at room temperature for 50 min and protected from light. The cell nuclei were stained with Sigma DAPI (Servicebio, Wuhan, China). The sections were observed under a fluorescence microscope (Nikon, Tokyo, Japan), and images were captured. The mean pixel fluorescence intensity was analyzed using ImageJ (version 6.0) (National Institutes of Health). A region of the same size was selected, the images of different channels were segmented, and then the red channel fluorescence images were converted to 8-bit black and white images. The average grey value was measured under a certain threshold, representing the average fluorescence intensity. Mean fluorescence intensity (Mean) = IntDen of the area/Area. Three independent biological replicates were performed in each group.

### 2.7. Histopathological Assessment

After sacrificing the mice, the left testis of each mouse was fixed in Bouin solution at 25 °C for 12 h. The fixed tissues were paraffin-embedded and sectioned, and then the sections were stained using hematoxylin and eosin to detect pathological damage. The sections used for HE staining were each 4 µm thick, and 3 sections were made for each sample.

### 2.8. Electron Microscopy

Fresh testicular tissue was immediately put into 3% glutaraldehyde and soaked for 4 h. After the end of soaking, the tissue was rinsed three times for 15 min each using 0.1 M phosphate buffer PB (PH 7.4) and then fixed for 2 h at room temperature (20 °C) using 1% osmium acid–0.1 M phosphate buffer PB (PH 7.4). After dehydration, infiltration, and embedding, ultrathin sections were made using an ultratome slicing 60–80 nm. Using 2% uranyl acetate and citrate staining, 15 min sections were dried at room temperature overnight, and the ultrastructure of the seminiferous epithelium was observed under a transmission electron microscope. The sections used for electron microscopy were 60–80 nm thick, and 3 sections were made for each sample.

### 2.9. Metagenomic Sequencing

Total genomic DNA was extracted from 0.5 g of stool material using the E.Z.N.A.^®^ Soil DNA Kit (Omega Bio-tek, Norcross, GA, USA) according to the manufacturer’s instructions. The concentration and purity of the extracted DNA were determined using TBS-380 and NanoDrop2000, respectively. DNA quality was checked on a 1% agarose gel. The DNA extract was fragmented to an average size of approximately 400 bp using Covaris M220 (Gene Company Limited, Hong Kong, China) for paired-end library construction. The paired-end library was constructed using NEXTFLEX Rapid DNA-Seq (Bioo Scientific, Austin, TX, USA). Paired-end sequencing was performed on Illumina Novaseq 6000 (Illumina Inc., San Diego, CA, USA) at Majorbio Bio-Pharm Technology Co. (Shanghai, China) using the NovaSeq 6000 S4 Reagent Kit according to the manufacturer’s instructions (www.illumina.com, accessed on 1 January 2023). The raw sequences were optimized for splitting, mass shearing, and contamination removal. The optimized sequences were then used for splice assembly and gene prediction, and the resulting genes were annotated for species and function as well as classified, including NR, EggNOG, KEGG, and so on, for subsequent analysis.

### 2.10. Liquid Chromatography–Mass Spectrometry (LC-MS) Untargeted Metabolomics

Metabolites were extracted from mouse blood for metabolomics analysis following the extraction methods described in previous studies [23]. A pooled quality control (QC) sample was prepared by mixing equal volumes of all samples, and the QC sample was processed and tested in the same way as the analyzed samples. This helped to represent the entire sample set, which was injected at regular intervals (every 5–15 samples) to monitor the stability of the analysis. LC-MS/MS analysis: The LC-MS/MS analysis of the sample was conducted on a Thermo UHPLC-Q Exactive system equipped with an ACQUITY HSS T3 column (100 mm × 2.1 mm i.d., 1.8 μm; Waters Corporation, Milford, MA, USA) at Majorbio Bio-Pharm Technology Co. Ltd (Shanghai, China). The MS conditions are described in the study by Zhan [24].

Equal volumes of metabolites from all samples were mixed to prepare QC samples, which were processed and analyzed similarly to the test samples. One QC sample was inserted with every eight test samples to monitor the stability of the analysis. A total of 4 QC samples were analyzed.

### 2.11. Statistical Analysis

The primary data were statistically analyzed using SPSS 26.0. Comparisons between groups were performed using one-way analysis of variance (ANOVA) with LSD when variances were congruent and Dunnett’s *t* test when variances were not congruent. Abnormal data were analyzed using the Kruskal–Wallis H test. Trends in body weight changes in the mice were analyzed using repeated measures ANOVA. A two-sided test *p* < 0.05 was considered a statistically significant difference.

Gut microbiology data were analyzed on a free online platform on the Majorbio Cloud Platform (www.majorbio.com, accessed on 1 January 2023). A catalog of non-redundant genes was constructed by filtering and assembling. The best-hit taxonomy of non-redundant genes was obtained by aligning them against the NCBI NR database by DIAMOND (http://ab.inf.uni-tuebingen.de/software/diamond/, version 2.0.11, accessed on 1 January 2023) with an e-value cutoff of 1 × 10^−5^ [25]. Based on the taxonomic and functional annotations and the abundance profiles of the non-redundant genes, differences were analyzed at each taxonomic, functional, or gene level using the Kruskal–Wallis test. Using SOAPaligner (https://github.com/ShujiaHuang/SOAPaligner, version soap2.21 release, accessed on 1 January 2023) software [26] (http://soap.genomics.org.cn/, accessed on 1 January 2023), the high-quality reads of each sample were compared to the set of non-redundant genes individually (default parameter: 95% identity), and the abundance information of the genes in the corresponding samples was counted. The abundance was calculated using Reads Per Kilobase Million (RPKM) [27], i.e., the number of reads per million sequences in one thousand bases per gene in the comparison.

LC-MS raw data were imported into the metabolomics processing software Progenesis version QI 3.0 (Waters Corporation, Milford, MA, USA), matched against the metabolic public databases HMDB (http://www.hmdb.ca/, accessed on 1 January 2023) and Metlin (https://metlin.scripps.edu/, accessed on 1 January 2023) and Meggie’s own libraries to obtain metabolite information. The data matrix after the library search was uploaded to the Meggie cloud platform (https://cloud.majorbio.com, accessed on 1 January 2023) for analysis. Firstly, the data matrix was preprocessed, and the preprocessing steps are described in Supplementary Material (Supplementary Material S1.3). Secondly, the ropls package in R (Version 1.6.2) was used to perform principal component analysis (PCA) and orthogonal least partial squares discriminant analysis (OPLS-DA) on the preprocessed data matrix, and the stability of the model was assessed using 7 cycles of cross-validation. The metabolites with variable importance in the projection (VIP) > 1, *p* < 0.05, were determined as significantly different metabolites based on the VIP obtained by the OPLS-DA model and the *p*-value generated by the student’s *t* test. Differential metabolites between two groups were mapped into their biochemical pathways through metabolic enrichment and pathway analysis based on the KEGG database (https://www.kegg.jp/kegg/pathway.html, accessed on 1 January 2023). The Python package scipy.stats was used to perform pathway enrichment analysis, and Fisher’s precision probability test was used to obtain the most relevant experimental treatments for the Biological pathways. After constructing gene sets based on taxonomic and functional annotations and abundance profiles of non-redundant genes, differential analyses were performed at each taxonomic, functional, or gene level using the Kruskal–Wallis test.

## 3. Results

### 3.1. Drinking Water Exposure to PS-NPs Induced Reproductive Dysfunction in Male Mice

#### 3.1.1. Body Weight and Changes in Testicular Organ Coefficients after Drinking Exposure to PS-NPs

During the exposure, one mouse from each of the 20 nm–0.1 mg/L and 20 nm–5 mg/L groups died before the end of the experiment. Based on their observations, the two male mice showed decreased appetite, activity, and weight loss before death.

**Figure 2:**
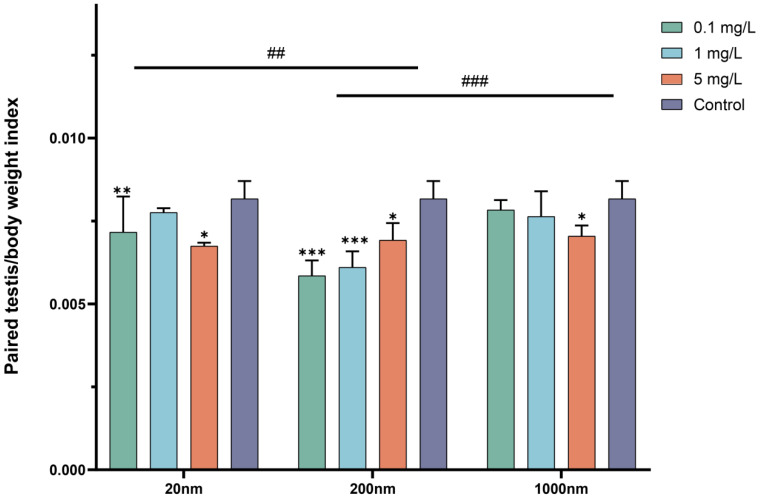
Body weight and changes in the organ coefficient after drinking exposure to PS-NPS. Note: “#” represents comparisons among exposure groups of different particle sizes, “##” is *p* < 0.01 and “###” is *p* < 0.001. “*” represents comparisons between exposure groups of different particle sizes and the control group. “*” is *p* < 0.05, “**” is *p* < 0.01, and “***” is *p* < 0.001.

The body weight of the mice in all groups increased over time. The observations showed that the body weight of the mice in the exposure groups was higher than that of the control group, but the difference was insignificant (see Supplementary Materials Figure S1). Significant differences in the organ coefficients of mouse testicular tissues were found among the exposure groups with different particle sizes at the end of the exposure (*p* < 0.001) (Figure 2).

#### 3.1.2. Changes in Sperm Quality and Sex Hormones after Drinking Water Exposure to PS-NPs

**Figure 3:**
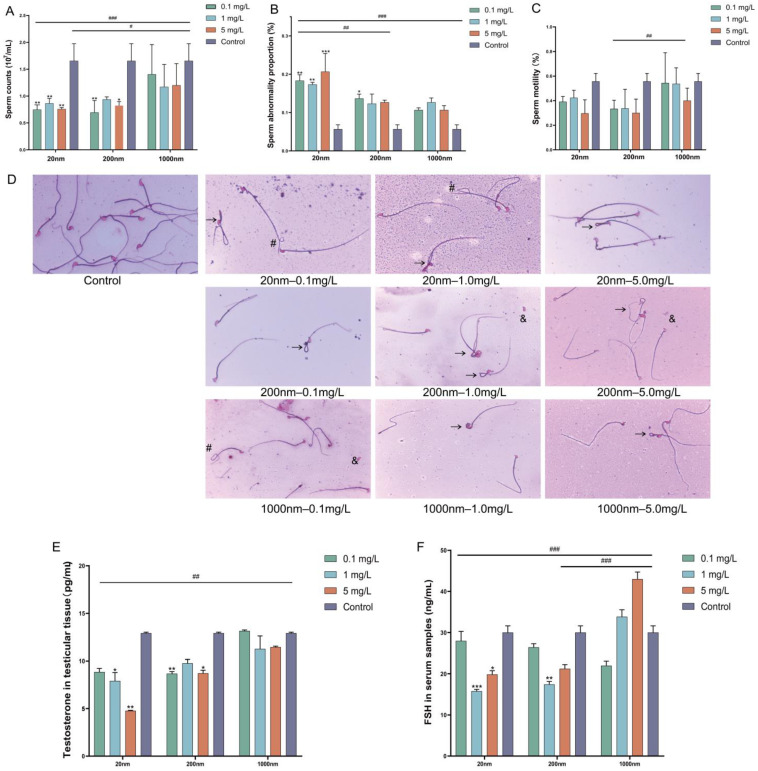
Changes in sperm parameters and sex hormones after drinking water exposure to PS-NPs. Note: Changes in sperm counts (**A**), sperm abnormality proportion (**B**), and sperm motility (**C**) after exposure. Representative images of sperm with Eosin staining (**D**). The pentagram indicates sperm with normal morphology, while “&” indicates tailless, “→” indicates cervical folding, and “#” indicates a curly tail. The level of testosterone in testicular tissue (**E**) and FSH in serum (**F**). “#” represents comparisons among exposure groups of different particle sizes, “*” represents comparisons between exposure groups of different particle sizes and the control group. “*/#” is *p* < 0.05, “**/##” is *p* < 0.01, “***/###” is *p* < 0.001.

After drinking water exposure to microplastics, the male mice showed a significant decrease in total sperm count and a significant increase in the proportion of sperm abnormality (Figure 3A,B). Although a decrease in sperm motility was observed in the exposure group in this study, no significant difference was found between the exposure group and the control group (Figure 3C). The malformed spermatozoa mainly showed cervical folding, curly tails, and tailless malformations (Figure 3D). The testosterone level in testicular tissue (Figure 3E) and the FSH level (Figure 3F) in blood were significantly decreased in the exposure groups compared with the control group. The above indexes were most significantly different in the 20 nm exposure group (*p* < 0.05).

#### 3.1.3. Changes in Inflammatory Factors and Oxidative Stress in Testicular Tissue after Exposure

**Figure 4:**
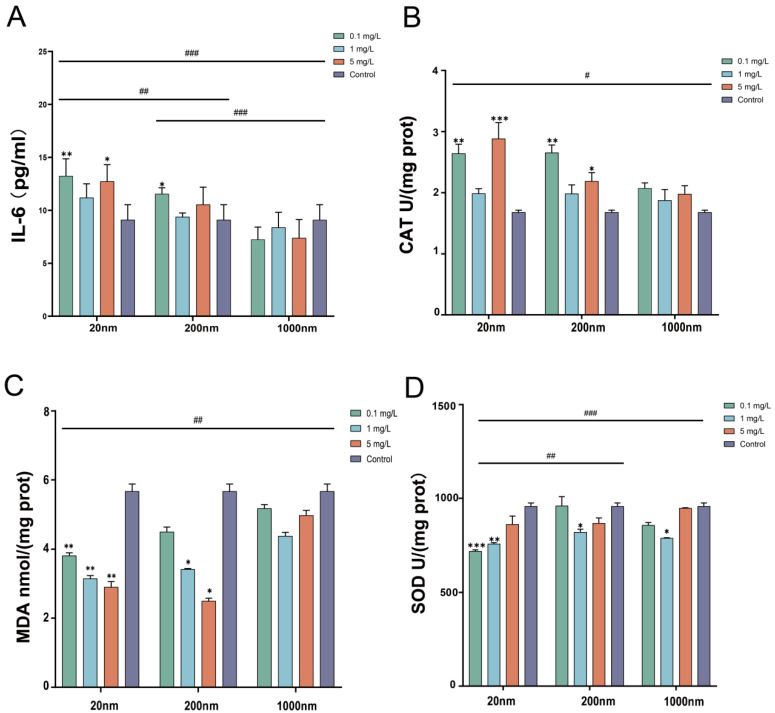
Changes in inflammatory factors and oxidative stress in testicular tissue after exposure. Note: Changes in inflammatory factors ((**A**): IL-6) and oxidative stress ((**B**): CAT, (**C**): MDA, and (**D**): SOD)) in testicular tissue after drinking exposure to PS-NPs. “#” represents comparisons among exposure groups of different particle sizes, “*” represents comparisons between exposure groups of different particle sizes and the control group. “*/#” is *p* < 0.05, “**/##” is *p* < 0.01, and “***/###” is *p* < 0.001.

Figure 4 shows that free drinking water exposure to PS-NPs caused an increase in the expression of IL-6 and CAT levels (*p* < 0.05) and a decrease in the expression of MDA and SOD levels in the testicular tissues of mice in the exposure groups compared with the control group (*p* < 0.05). However, no significant changes in the levels of IL-1β, TNF-α, or GSH-PX were found in the exposure groups (see Supplementary Material Figure S2). Moreover, we found significant differences in the expression of IL-6, CAT, MDA, SOD, and GSH-PX levels among the exposure groups with different particle sizes (*p* < 0.05) (Figure 4).

#### 3.1.4. IL-6 and TNF-α Expression in Mice Testes Detected by Immunofluorescence

**Figure 5:**
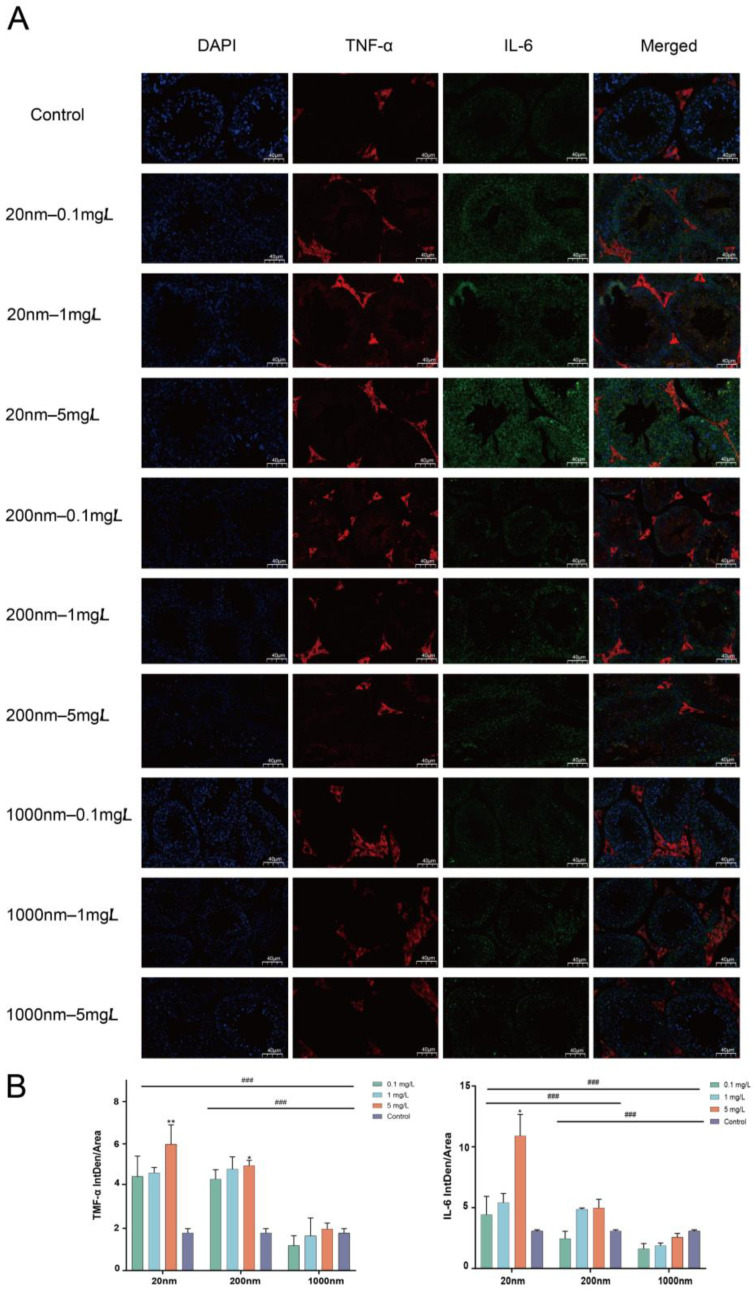
Immunofluorescence assay for TNF-α and IL-6 expression in mouse testes. Note: Immunofluorescence staining of testicular tissue, with TNF-α fluorescence expression in red, IL-6 fluorescence expression in green, and DAPI nuclear staining in blue (**A**). Quantitative detection and analysis of testicular tissue immunofluorescence staining (**B**). “#” represents comparisons among exposure groups of different particle sizes, “###” is *p* < 0.001. “*” represents comparisons between exposure groups of different particle sizes and the control group. “*” is *p* < 0.05 and “**” is *p* < 0.01.

The immunofluorescence results show significant expression of IL-6 and TNF-α in mouse testicular tissues after exposure to PS-NPs (Figure 5A). After quantitative analyses, it was found that there was a significant difference in the expression of IL-6 and TNF-α in the exposure groups with different particle sizes (*p* < 0.05) (Figure 5B).

#### 3.1.5. Changes in Testicular Histological Structure after Exposure to NPs in Drinking Water

**Figure 6:**
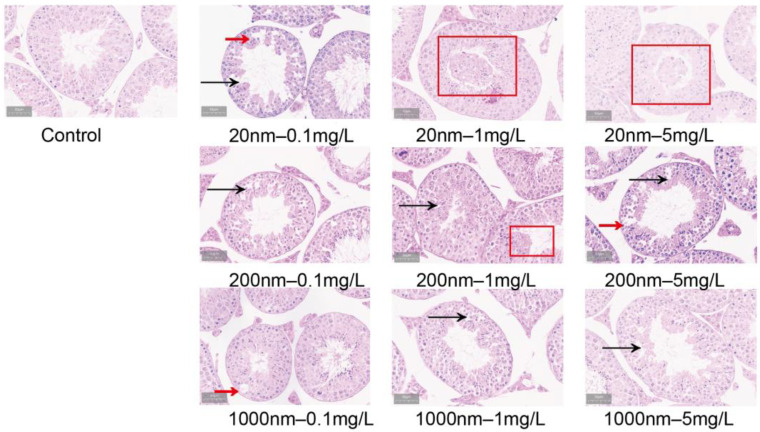
HE staining of mouse testicular tissue (50 μm). Note: black arrows indicate sparse structure, red arrows indicate vacuolization of testicular tissue, and red boxes indicate detached germ cells.

**Figure 7:**
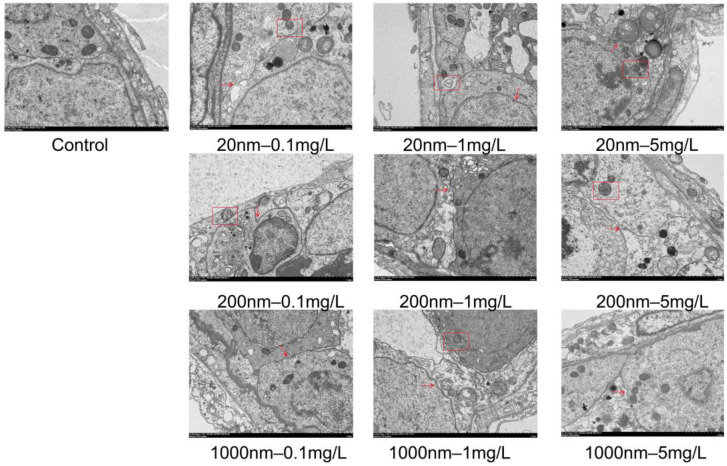
Electron microscopic structure of mouse tissue (2 μm). Note: Red arrows indicate the blood–testis barrier, and red boxes indicate structurally disrupted mitochondria.

As shown by HE staining, the control group had a regular and tight arrangement of spermatogenic cells and abundant mesenchymal cells, while the histopathological structures of testicular tissues in the groups of mice exposed to PS-NPs were significantly altered, showing a disordered arrangement of spermatogenic cells, intra-epithelial vacuolization, and exfoliated germ cells (Figure 6). Electron microscopy showed the disassembly of blood–testis barrier (BTB) structural cells in the testicular tissue of the exposure groups of mice, disruption of continuity, and abnormalities in some mitochondrial structures (Figure 7).

### 3.2. Macrogenomic Alterations after Drinking Water Exposure to PS-NPs

#### 3.2.1. Macrogenomic Annotation Information Statistics

Based on macrogenomic annotation information, it is known that mice microbiota is structured as follows: domain: five, kingdom: 14, phylum: 214, class: 411, order: 812, family: 1625, genus: 4319, and species: 17,378.

**Figure 8:**
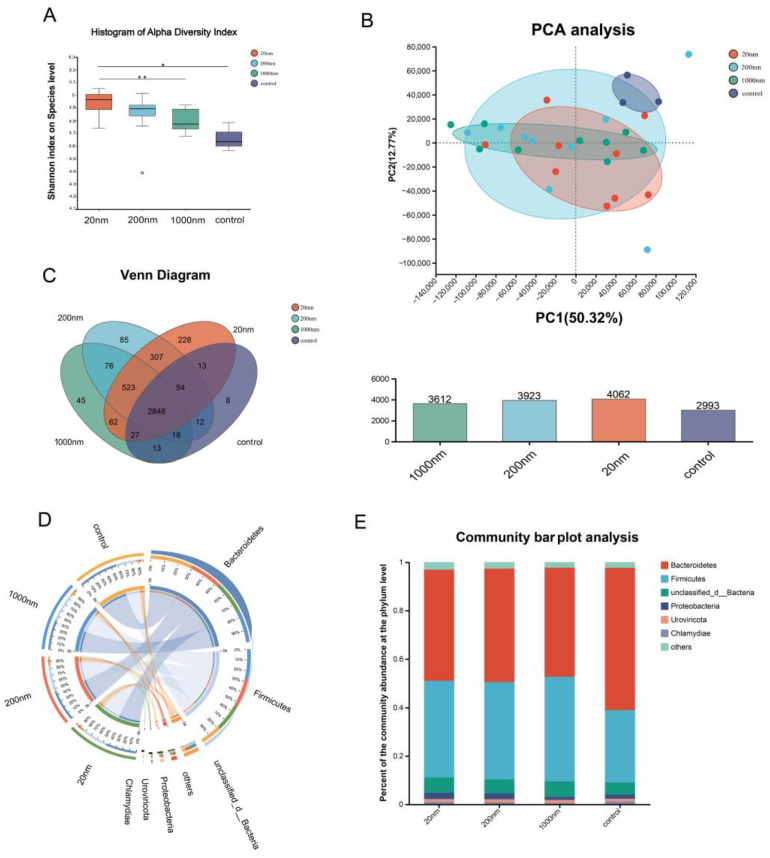
Changes in gut NR-annotated species diversity and community species composition. The comparison of the Alpha diversity of NR genes in different exposure groups at the species level (**A**). “*” is *p* < 0.05 and “**” is *p* < 0.01. The PCA analysis of NR genes in different exposure groups at the phylum level (**B**). Venn analysis of NR genes in different exposure groups at the genus level (**C**). Circos plot at the phylum level for difference exposure groups (**D**). Community bar plot analysis at the phylum level (**E**).

Figure 7 shows that drinking water exposure to PS-NPs caused an increase in the Alpha diversity of gut microbiota in mice, with the highest Alpha diversity in the 20 nm exposure group (Shannon index) (Figure 8A), details of which are provided in the Supplementary Materials (see Supplementary Material Figure S3). Beta diversity was analyzed using principal component analysis (PCA), which showed that drinking water exposure to PS-NPs significantly affected the NR-annotated species composition of the gut microbiota in the mice (Figure 8B). Venn analysis showed that the 20 nm group had the most specific species, and the 1000 nm group shared the most species with the control group (Figure 8C). The Circos plot showed that the phylum level of *Bacteroidetes* and *Firmicutes* were the dominant bacterium in each group (Figure 8D). The community composition changed in each exposure group compared with the control group, such as a decrease in the abundance of *Bacteroidetes* and an increase in the abundance of *Firmicutes* (Figure 8E).

#### 3.2.2. Changes in the Abundance of Gut Microbes Following Drinking Water Exposure to Polystyrene

**Figure 9:**
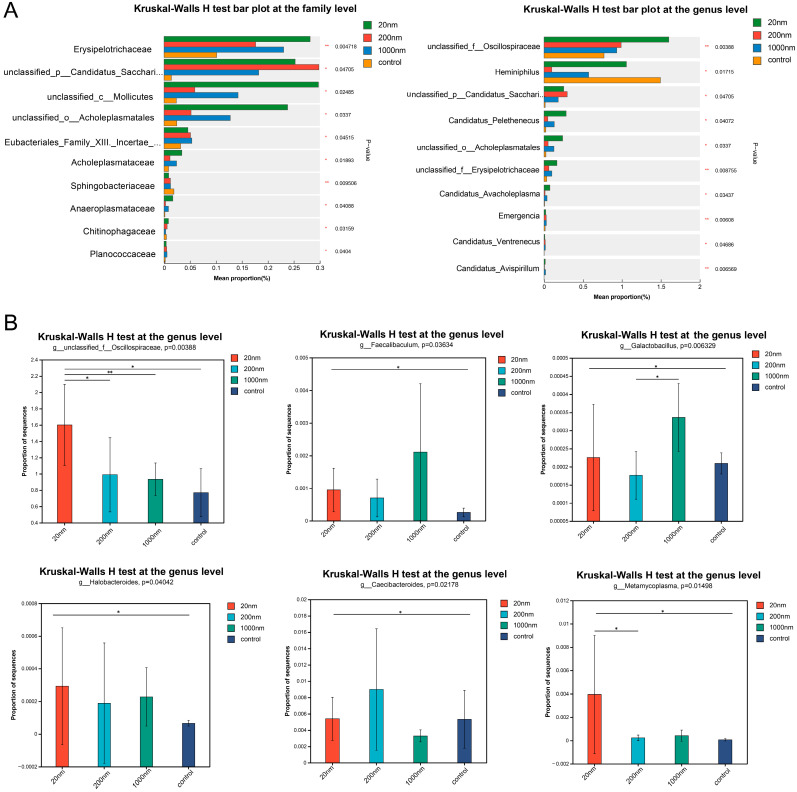
Changes in the abundance of gut microbes. The differences in gut microbial species at the family level (**left**) and genus level (**right**) among the groups (**A**); The gut microbes associated with male reproductive function at the genus level (**B**). “*” is *p* < 0.05 and “**” is *p* < 0.01.

The differential analysis of gut microbial abundance showed that a total of 109 families and 299 genera differed among the exposure and control groups (*p* < 0.05) (Figure 9A). Some of the gut microbiota was found to differ among the exposed and control groups (*p* < 0.05) (Figure 9B).

#### 3.2.3. Correlation Analysis between Gut Microbial Abundance and Testicular Tissue Levels of Testosterone, Inflammation, and Oxidative Stress

**Figure 10:**
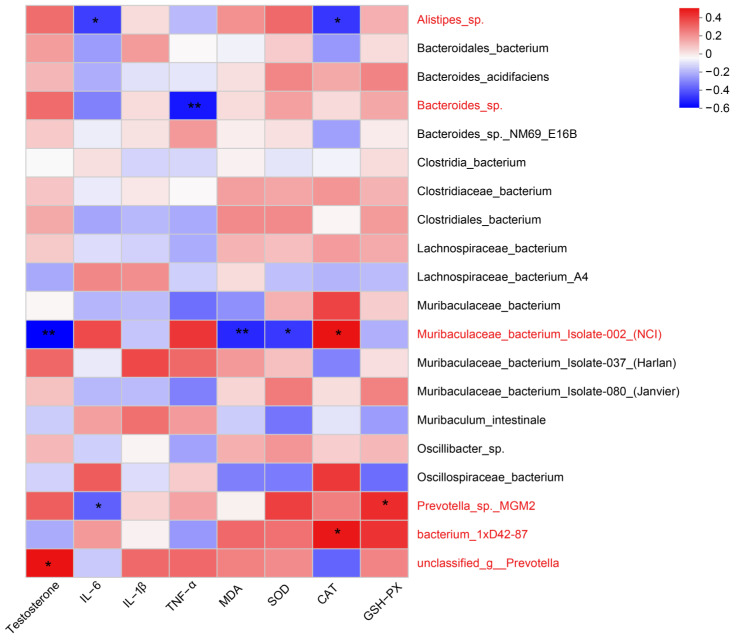
Correlation analysis heatmap showing gut microbial abundance and testicular tissue levels of testosterone, inflammation, and oxidative stress. Note: R-values are shown in different colors in the figure. “*” is *p* < 0.05 and “**” is *p* < 0.01.

The heatmap plot analysis revealed correlations among gut microbial abundance and testosterone, IL-6, MDA, SOD, CAT, and GSH-PX at the genus level (Figure 10). The results demonstrated a correlation between changes in gut microbial abundance levels and male reproductive function in mice.

### 3.3. LC-MS Untargeted Metabolomics Analysis

#### 3.3.1. Metabolite Annotation Information

**Figure 11:**
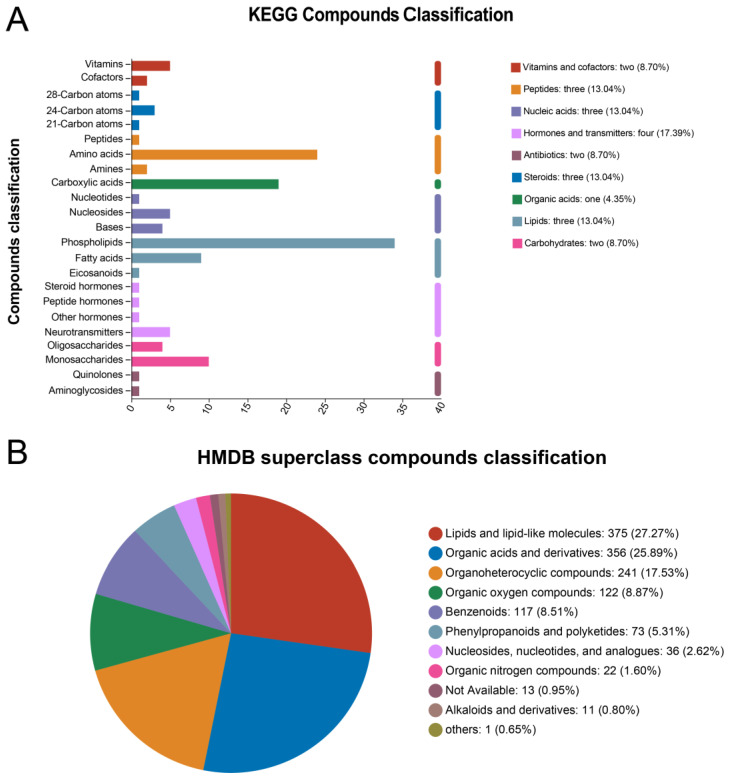
Metabolite annotation information and composition.

Table 1 shows the total number of ions detected and the metabolite identification statistics in the serum samples from the groups. The identified metabolites were compared to the KEGG Compound and HMDB 4.0 databases to obtain the metabolite classification profiles. The primary classification showed that hormones and transmitters were the most represented compounds in the KEGG Compound classification, and the secondary classification showed that phospholipids were the most represented compounds (Figure 11A). Lipids and lipid-like molecules were the most annotated compounds in the HMDB 4.0 database (Figure 11B). Linear discriminant analysis (LDA) revealed the microbial taxa that contributed significantly to the differences among groups, with the 20 nm exposure group contributing the most subgroup differences (see Supplementary Material Figure S4).

#### 3.3.2. KEGG Differential Metabolite Enrichment Analysis

**Figure 12:**
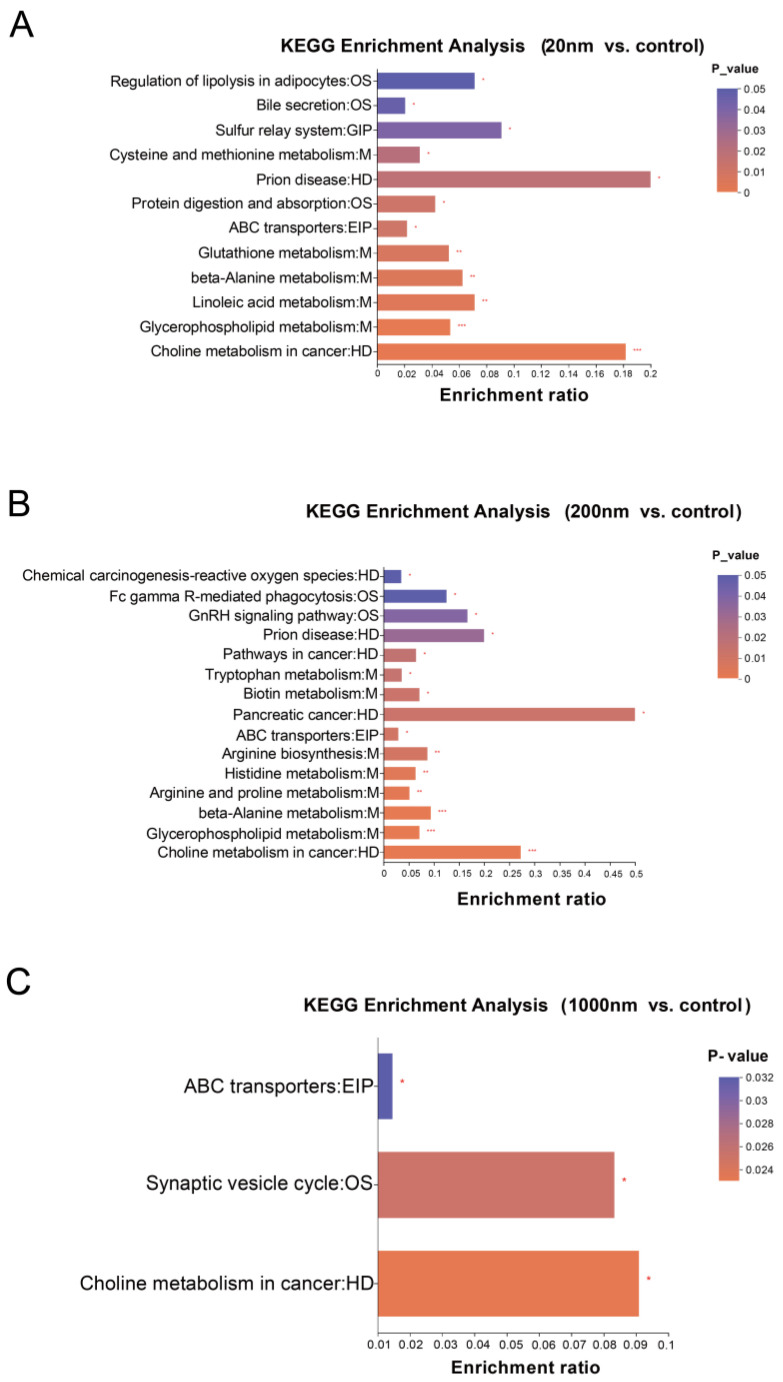
KEGG differential metabolite enrichment analysis. Note: 20 nm vs. control (**A**), 200 nm vs. control (**B**), 1000 nm vs. control (**C**). The figure is the KEGG enrichment analysis plot, where the horizontal coordinate indicates the pathway name and the vertical coordinate indicates the enrichment rate. “*” is *p* < 0.05, “**” is *p* < 0.01, and “***” is *p* < 0.001.

The exposure group showed significant changes in metabolites and altered the enrichment of metabolites in a number of important metabolic pathways compared with the control group. In addition, the 1000 nm group had the least altered metabolic pathways compared with the control group, of which there were only three. In contrast, alterations in more metabolic pathways related to male reproductive function were found in the 20 nm and 200 nm exposure groups, which mainly focused on amino acid metabolism, glutathione metabolism, and protein metabolism pathways, such as Linoleic acid metabolism, Tryptophan metabolism, and so on (Figure 12).

**Figure 13:**
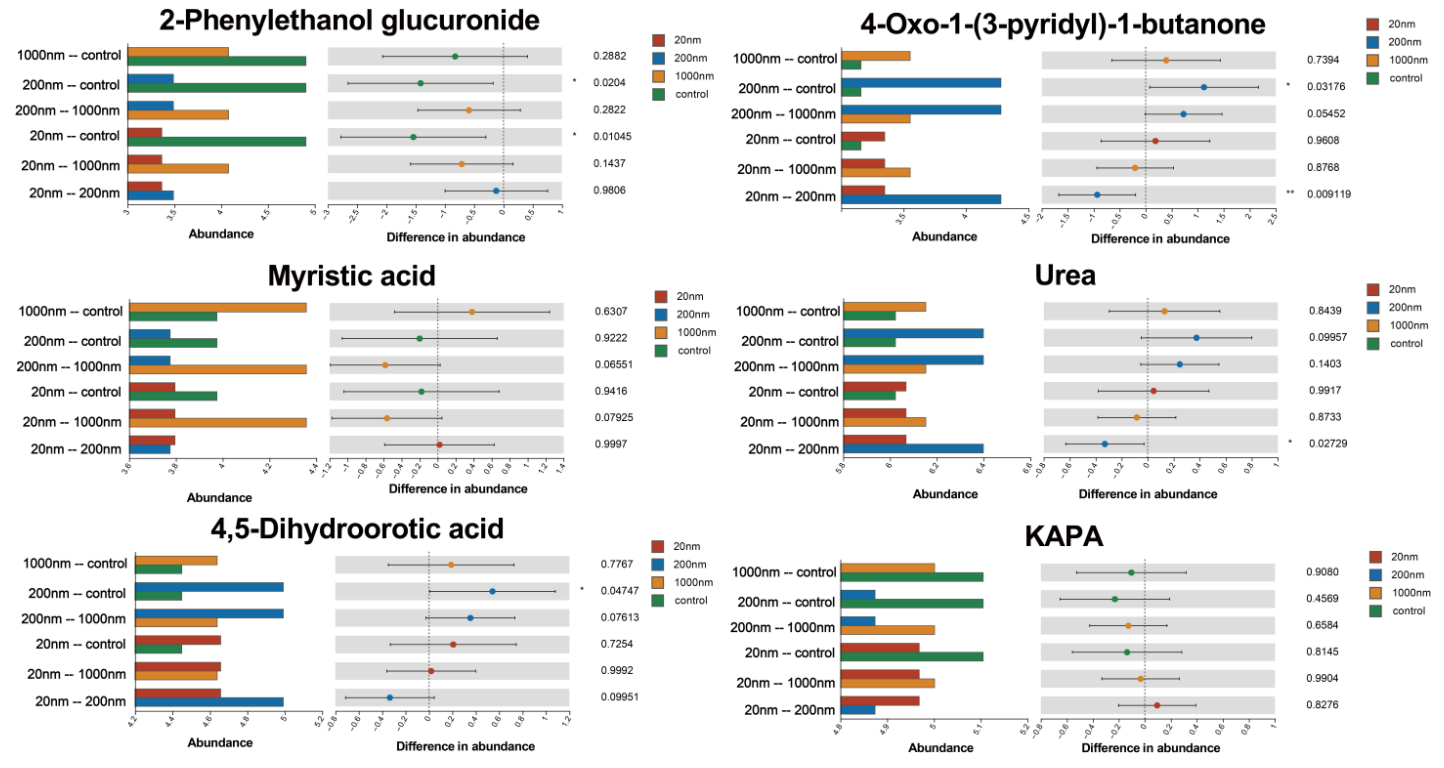
Differential metabolite analysis. Note: The horizontal coordinate of the left bar graph indicates the average relative abundance of a metabolite in different subgroups, the vertical coordinate indicates the subgroup category in a two-by-two comparison, and different colors indicate different subgroups. The middle area is the confidence interval set, the value corresponding to the dot indicates the difference in the average relative abundance of the metabolite in the two subgroups, the color of the dot is shown as the color of the subgroup whose metabolite abundance accounts for a larger proportion of the metabolite abundance, and the I-type intervals on the dots are the differences between the upper and lower values. “*” is *p* < 0.05 and “**” is *p* < 0.01.

The differential metabolite analyses showed that drinking water exposure to PS-NPs led to the emergence of 109 metabolites that differed among the exposure and control groups. In addition, we identified differential metabolites, such as corticosterone, that have been shown to be associated with male reproductive dysfunction in previous studies (Figure 13).

## 4. Discussion

In this study, we established an animal model of PS-NP exposure in free drinking water to investigate the effects of different particle sizes and concentrations of PS-NP on the reproductive functions of male mice and the potential mechanisms. The results showed that male mice exposed to PS-NPs exhibited impaired reproductive functions, including structural damage of testicular tissues, decreased sperm quality, and decreased sex hormone levels. Moreover, exposure to PS-NPs also altered the oxidative stress status of testicular tissues and increased the expression of inflammatory factors in testicular tissues. In addition, the present study identified gut microbiota disorders and metabolic dysregulation, including increased microbial diversity, altered community structure, differential metabolite alterations, and significant enrichment in metabolic pathways. Notably, the particle size of PS-NPs is an essential factor affecting reproductive impairment in male mice.

### 4.1. Exposure to PS-NPs Causes Reproductive Impairment in Male Mice

Numerous studies have shown that NPs have become important environmental pollutants that pose potential hazards to human health [28,29,30]. Because of their unique physical properties, such as their small size and large specific surface area, NPs are more likely to pass through biological barriers in the body and enter cells, posing a health risk to organisms [28]. Previous studies have demonstrated that NPs can pass through the liver barrier and cause hepatotoxic damage [31], pass through the blood–brain barrier and increase the incidence of neurodegenerative diseases [32]; pass through the placental barrier and pose a health threat to the offspring [33]; and pass through the intestinal barrier and cause intestinal dysfunction and gut microbiota disorders [34]. However, in recent studies, it has been shown that NPs may also cause reproductive toxicity through the BTB, inhibiting spermatogenesis and affecting sperm quality [35]. Recent studies have shown that MNPs can also cause adverse effects at the cellular level, including oxidative stress [29], sperm DNA damage [36], and damage to the hypothalamus–pituitary–testicular (HPT) axis [37], which induces disruption of BTB structures in testicular tissues and testicular inflammation in several key pathways, leading to impaired reproductive function in mammals [38]. However, at this stage, there is still some controversy about the biological mechanisms of impaired male reproductive function caused by NPs [19,39].

Testicular tissue is an important site for sperm production in male animals and maintains normal male reproductive function [40]. A review study showed that NPs may reduce male fertility by inducing inflammation and oxidative stress in testicular tissues, thereby leading to altered BTB function, including reduced sperm viability and increased malformation rates [38]. In the present study, we similarly found that the organ coefficients of testicular tissues of mice in all PS-NP exposure groups were significantly lower than those of the control group, suggesting that drinking water exposure to PS-NPs may lead to atrophy or degenerative changes in testicular tissues of mice. Moreover, it was found that the sperm quality of the mice in the exposure groups was significantly decreased, and the sex hormone level was also significantly lower than that of the control group. Further, the morphological structure of mouse testicular tissue was observed by HE staining and electron microscopy, and the results showed that some spermatocytes were detached into the lumen and the continuity of BTB structure was interrupted. Taken together, the PS-NPs that entered the gastrointestinal tract of the mice via drinking water exposure may have crossed the intestinal barrier into the bloodstream and entered the testicular tissue cells via the BTB, resulting in impaired reproductive function. In addition, the results of this study showed that the IL-6 level in the testicular tissue of the mice in the exposure groups was significantly increased compared with that of the control group, which proved that the testicular tissue produced a certain inflammatory response under exposure to PS-NPs. And the results also found that the expression of CAT in the testicular tissues of the mice in the exposure group increased, and the expression of MDA and SOD decreased. Taken together, the changes in these indexes proved that exposure to PS-NPs caused oxidative stress in mouse testicular tissues. Previous studies have found that the development of testicular inflammation is associated with an increased rate of sperm deformity [16,22], atrophy, detachment, and apoptosis of spermatocytes in testicular tissue [22]. Xie’s study demonstrated that oxidative stress and the activation of the P38 MAPK signaling pathway due to MPs were the main factors contributing to sperm abnormalities, reduced counts, and activity in mice [41].

### 4.2. Exposure to PS-NPs Causes Reproduction-Associated Gut Microbiological Disturbances

Several studies have shown that the potential toxicity of NPs on male reproductive function is inextricably linked to gut microbial homeostasis [18,19]. Oral exposure is one of the most important ways that NPs enter an organism, so the gastrointestinal tract is considered to be the earliest exposed organ, and NPs entering the gastrointestinal tract may come into contact with intestinal epithelial cells and interact with the host gastrointestinal tract at the cellular level [42]. Although no change in the type of dominant bacteria was found in this study, the relative abundance level of the dominant bacteria in the exposure groups was altered compared with the control group, suggesting that PS-NP drinking water exposure may have led to the dysbiosis of the gut microbiota in the male mice. Previous studies have also found that oral exposure to NPs significantly altered the abundance level of intestinal microbiota and induced gut microbiota dysbiosis and related metabolic disorders [20]. In addition, in the present study, we found that exposure to PS-NPs resulted in altered oxidative stress activity-related indexes and increased expression of inflammatory factors in the testicular tissues of the mice in the exposure groups, as well as an increase in *Oscillospiraceae*, *Fecalibaculum*, and other bacteria in the gut of the mice in the exposure groups. As the largest immune organ, the intestine attenuates the organism’s chronic inflammatory response by regulating the abundance of various microorganisms and metabolic pathways when disturbed by exogenous factors [43,44]. Previous studies have found that the gut can produce large amounts of short-chain fatty acids by increasing the number of short-chain fatty acid-producing bacteria, which in turn reduces the body’s inflammatory response by modulating immune cell chemotaxis, regulating metabolism, inhibiting the metabolic transport of harmful substances such as ethanol, and lowering the concentration of lipopolysaccharides in the blood [45,46]. Therefore, we suggest that changes in the gut microbiota may be one of the compensatory mechanisms for the in vivo inflammatory response induced by PS-NPs and that the gut microbiota may be an important pathway to repair or reduce inflammation in testicular tissue.

The Alpha diversity of the gut microbiota has been correlated with male reproductive function, with higher Alpha diversity representing a more complex and stable composition of the gut microbiota, which enhances resistance and adaptability to external disturbances and favors host health. However, recent studies have shown that Alpha diversity is negatively correlated with total testosterone levels [47]. Although testosterone is an important hormone that promotes the formation of male reproductive organs and supports male reproductive function, when the testosterone level decreases, it affects spermatogenesis [48]. In this study, changes in the relative abundance levels of dominant bacteria were detected in the PS-NP drinking water exposure groups compared with the control group. Also, some bacteria associated with male reproduction differed significantly among the groups. For example, at the phylum level, we found that *Bacteroidetes* were significantly reduced in all exposure groups; their reduction may induce metabolic endotoxemia, whereas excess endotoxin inhibits steroid hormone synthesis in mesenchymal cells and reduces pituitary luteinizing hormone drive, which in turn reduces testosterone production and leads to spermatogonial reduction [49]. Thus, NPs may influence male reproductive function through the gut–testis axis. In addition, in the present study, by correlating gut microbiota with inflammatory factors, oxidative stress indicators, and hormone levels in the mice, gut microbiota such as *Alistipes*_sp., *Bacteroides*_sp., and *Prevotella*_sp. were found to be significantly correlated with testosterone, inflammation, and oxidative stress levels in testicular tissues [21,50]. In conclusion, PS-NPs entering the gastrointestinal tract induce gut microbiota-associated changes in testicular tissue levels of sex hormones, inflammation, and oxidative stress, leading to testicular dysfunction.

### 4.3. Exposure to PS-NPs Causes Reproduction-Related Metabolic Disorders

During colonization and reproduction, the gut microbiota and its host co-metabolize and produce a range of metabolites that directly or indirectly affect host health [51]. NPs are difficult for the human body to metabolize and absorb, and those that are not excreted accumulate in the body and, when above a certain level, can cause varying degrees of damage to the digestive tract and cells, triggering local inflammation and damaging the immune system. In particular, some of the chemicals contained in NPs can easily disrupt the body’s secretion system, leading to diabetes, obesity, and other diseases. Under laboratory conditions, Teng conducted zebrafish experiments using PS-NPs and showed that PS-NP exposure not only altered the gut microbiota of zebrafish but also led to changes in 42 metabolites related to neurotransmission [52]. Chen’s study similarly demonstrated that the exposure of mice to PS-MPs and PS-NPs altered ABC transporter, Aminoacyl-tRNA biosynthesis, amino acid biosynthesis, bile secretion, and other metabolic pathway enrichment [53]. The results of the present study similarly identified metabolic disturbances induced by PS-NP exposure. There were several metabolic pathways significantly enriched in the exposure groups compared with the control group, such as ABC transporter, choline metabolism in cancer, and so on. There were 100 metabolites that differed among the exposure and control groups.

Metabolite production and consumption are essential for maintaining normal reproductive function. It has been shown that alterations in some metabolic pathways affect the physiological function of testicular tissues and impair sperm formation and excretion [54]. The results of the present study suggest that some metabolites related to reproductive function were significantly different among the exposure and control groups, such as myristic acid, spermidine, and KAPA. Among them, myristic acid has been shown to significantly alleviate neuropathy caused by metabolic diseases and protect testicular tissues from dysfunction induced by conditions such as diabetes mellitus [55]. Spermidine, on the other hand, is involved in the process of glutathione and arginine metabolism, thereby modulating the function of testicular tissue [56]. In addition, spermidine, as a selective 5-hydroxytryptamine reuptake inhibitor, prolongs 5-hydroxytryptamine activity among neurons and improves male sexual dysfunction. Substances such as KAPA have also been shown to be involved in spermatogenesis in previous studies [57,58,59]. Furthermore, our results suggest that the exposure groups showed significant enrichment in a variety of metabolites associated with male reproduction, such as protein digestion and absorption, glycerophospholipid metabolism, glutathione metabolism, and ABC transporters. These changes cause male reproductive dysfunction through effects on energy metabolism, androgen synthesis, testicular tissue development, spermatogenesis and maturation, and prostaglandin metabolism. Such ABC transporters are a class of membrane transporter proteins that are widely found in organisms and use the energy generated by ATP hydrolysis to drive the transport of various molecules (e.g., ions, amino acids, sugars, phospholipids, and so on) across membranes. Studies suggest that certain ABC transporters may affect spermatogenesis, maturation, and motility [60]. Moreover, the glutathione metabolic pathway has also been shown to be one of the important factors affecting male infertility [61]. Reduced glutathione levels lead to increased levels of oxidative stress, which can disrupt the integrity of the sperm membrane during sperm production and lead to male infertility [62]. In summary, the metabolic dysregulation caused by free drinking water exposure to PS-NPs likely interfered with the normal reproductive function of the male mice and deserves attention. Combined with previous studies, we speculate that metabolic disorders caused by altered gut microbiota resulting from drinking water exposure to PS-NPs may contribute to reproductive dysfunction in male mice.

### 4.4. There Are Grain Size Differences in the Reproductive Damage Caused by PS-NPs

The results of the present study showed that the reproductive damage caused by PS-NPs of different particle sizes in male mice differed. Oxidative stress and inflammatory factors in testicular tissues were significantly different among the different particle size groups. This study showed that as the particle size of PS-NPs decreased, the reproductive function of the mice was more seriously affected. In the present study, we found that the sperm quality decreased more and the immune response was more severe in the 20 nm and 200 nm groups compared with the 1000 nm exposure group. Previous studies have also found such particle size differences in other tissues and organs [63]. Yu’s study found particle size dependence in the toxicity damage of NPs in zebrafish, and their study found that liver histopathological damage was more severe as the particle size of MPs decreased [64]. Interestingly, we found a positive correlation among the Alpha diversities of gut microorganisms resulting from exposure to PS-NPs of different particle sizes: the smaller the particle size, the more diverse the gut microorganisms. This may be related to the increase in pathogenic microorganisms resulting from exposure to NPs in the gut. In the present study, it was found that the abundance level of pathogenic microorganisms such as mycoplasma was significantly higher in the 20 nm exposure group as compared with the other two groups. Similarly, metabolomics analyses showed that nanoscale PS-NPs resulted in significant enrichment of more metabolic pathways. Possible reasons for the particle size dependence of NPs include the following: (1) NPs have a very high specific surface area because of their small size, which makes the interaction among NPs, tissue cells, and harmful environmental substances in the organism more significant. (2) Small-sized NPs are more likely to pass through tissue gaps and enter cells and biological barriers, thereby triggering more biological effects. For example, scholars have demonstrated that NPs are more likely than MPs to pass through cell membranes and reach the interior of cells, where they interact with intracellular proteins and DNA, leading to cellular dysfunction [63]. (3) The particle size of NPs also affects their migration and transformation in the environment; the small size of NPs makes it easier for them to adsorb into other substances and migrate and makes it easier for them to react with other substances, including adsorption, aggregation, and transformation, leading to co-toxicity effects.

### 4.5. Strengths and Limitations

In this study, we investigated the particle size effect of the toxicity injury of NPs by using different particle sizes of PS-NPs for exposure, and we used macrogenomics and metabolomics analyses to investigate the potential mechanism of injury, which improved the reliability of the results and avoided the issue of finding insufficient evidence for the results of a single study. In addition, by simulating real human exposure scenarios and using long-term low-dose chronic exposure, this study provides highly realistic and reliable results, which is important for assessing potential health risks. However, the present study did not find a concentration-dependent impairment of reproductive function by NPs in male mice. This result may stem from the fact that the present study used a low-concentration chronic exposure mode with free drinking water. In this mode, the toxic effects caused by the prolonged exposure of mice to low concentrations of NPs were not significant in terms of concentration differences. However, we still found reproductive function impairment in the exposure groups of mice when compared with the control group at the end of the exposure. So, this study demonstrated that chronic, cumulative impairments may be triggered by long-term exposure at low concentrations and that these impairments may not manifest themselves until after a longer exposure time or at a higher exposure dose. In addition, the small particle size of the PS-NPs used in this study is likely to be eliminated from the body through metabolism, resulting in a smaller amount of PS-NPs actually retained in the organism, which led to a non-significant difference in toxicity damage among the three concentration groups. Furthermore, since the indicators related to reproductive damage, metabolism, and gut microbiota analyzed in this study were all post-exposure, there is a need to expand the sample size in the future and set up further crossover experiments for reverse extrapolation to obtain more robust results.

## 5. Conclusions

With increasing NP pollution, the potential health hazards caused by NPs should not be underestimated. In this study, we investigated the effects of 20 nm, 200 nm, and 1000 nm PS-NPs on the reproductive function of male mice through chronic intervention using free drinking water exposure and analyzed the changes in macrogenome sequencing and metabolomics. The results showed that PS-NPs caused changes in the gut microbiota and metabolites related to reproduction, as well as abnormal alterations in the function of testicular tissues. We also found that there was a certain size-dependent reproductive toxicity of PS-NPs in male mice. The present study provides evidence for the reproductive toxicity hazards of NPs. To prevent further decline in male fertility levels in the future, there is a need to strengthen the avoidance of NP pollution in the future.

## Figures and Tables

**Table 1:** Total ion count and identification statistics table.

Ion Mode	All Peaks	Identified Metabolites	Metabolites in Library	Metabolites in KEGG
pos	4138	943	884	528
neg	3825	531	508	293

Note: (1) Ion mode: the ion mode of the substance detected by the mass spectrometer, mainly pos (positive ion mode) and neg (negative ion mode). (2) All peaks: the number of mass spectrometry peaks extracted by the software. (3) Identified metabolites: the number of metabolites finally identified through primary and secondary mass spectrometry data and searched in libraries (self-built libraries, Metlin, HMDB, etc.). (4) Metabolites in library: the number of metabolites annotated to public databases such as HMDB and Lipidmaps. (5) Metabolites in KEGG: the number of metabolites annotated to the KEGG database.

## Data Availability

All data collected or analyzed in this study are included in this published article and can be acquired from the corresponding author on reasonable request.

## Supplementary Materials

The following supporting information can be downloaded at: https://www.mdpi.com/article/10.3390/toxics12080531/s1, Figure S1: Body weight after drinking exposure to PS-NPs; Figure S2: Changes in inflammatory factors and oxidative stress in testicular tissue after exposure; Figure S3: Analysis of the alpha diversity of gut microbiota; Figure S4: LDA of Macrogenomic annotation; Figure S5: Imaging small animals in vivo.
