# Supplementary material for: Toxicity to the Male Reproductive System after Exposure to Polystyrene Nanoplastics: A Macrogenomic and Metabolomic Analysis
Source: Toxics. 2024 Jul 23;12(8):531. doi: 10.3390/toxics12080531 (PMC11360567; doi:10.3390/toxics12080531)
Supplement: Supplementary file 1 [file toxics-12-00531-s001.zip › toxics-3077606-supplementary.pdf]

Impairment of Reproductive Function and Corresponding Macrogenomic and Metabolomic Alterations in Male Mice Exposed to Polystyrene Nanoplastic Drinking Water

Supplementary materials

S1. Supplementary methods

S1.1. Step of ELISA kit

Although there are minor differences between the kits for different indicators, the basic steps of the ELISA kit are the same: (1). Encapsulation: dilute the antibody with carbonate encapsulation buffer to a protein content of 1–10 μg/ml. add 100 μl to each well of a polystyrene ELISA plate and leave overnight at 4 °C. The next day, discard the solution in the wells and wash with washing buffer 3 times, each time for 3 min. (This step can be omitted in commercial kits.) On the next day, discard the solution in the wells and wash with washing buffer for 3 times, 3 min each time.(Generally, the antibody is already encapsulated in the commercial kits, this step can be omitted). (2). Closure: add 200 ul of closure solution to each well and incubate at 37 ℃ for 1–2 h. (3). Wash: carefully remove the sealing membrane, put it into the plate washer and wash it for 3–5 times. The plate can also be washed manually: discard the liquid, add 300 ul of washing solution to each well, soak for 1-2min, pat dry on absorbent paper and repeat 3–5 times. (4). Spiking: add 100 μl of appropriately diluted sample to be examined into the above reaction wells which have been coated. (At the same time, do the blank wells, doubly diluted standard wells). (5).Warming: seal the plate with plate sealing film and incubate at 37 ℃ for 1–2 h. (6). Wash: same as step 3. (7). Antibody addition: add diluted biotinylated antibody working solution of 100 μl into each well. (8). Warming: seal the plate with plate sealing film and incubate at 37 ℃ for 1 h. (9).Wash: same as step 3. (10) Enzyme conjugate addition: add diluted enzyme conjugate working solution of 100 μl into each well. (11). Warming: add diluted enzyme conjugate working solution of 100 μl into each well. (12). Warming: After sealing the plate with sealing membrane, incubate at 37 ℃ for 30 min, avoiding light. (13). Wash: same as step 3. (14). Add colour-developing substrate: Add 100 μl of TMB substrate solution to each well, and react at 37 ℃ for 10–30 min, avoiding light, until a clear colour gradient appears in the wells of doubly diluted standards. (15). Terminate the reaction: Add 2M sulphuric acid to each well. The reaction was terminated by adding 100 μl of 2M sulfuric acid to each well, and the colour changed from blue to yellow. (16). Measurement of results: Within 10 min, the OD value of each well was measured at 450 nm on an enzyme marker after zeroing with a blank control well. (17). Calculation of ELISA results. Make a standard curve according to the concentration and OD value of the standard, and then calculate the sample concentration according to the standard curve equation.

S1.2. Small animal in vivo imaging experiments

In this study, in vivo fluorescence luminescence imaging experiments were performed using a small animal imager (manufacturer PerkinElmer LLC, model Lumina LT). Twelve male mice of 5-week-old size were used as the main experimental animals and were divided into three exposure groups and one control group. The three exposure groups were intervened with 20 nm, 200 nm and 1000 nm fluorescent microplastics (Servicebio, China). 0.1 ml of microplastic with fluorescent signal was made to enter into the mice by gavage. The mice are anaesthetised with ether, and after anaesthesia, they are placed into the imaging dark box platform. The software controls the lifting and lowering of the platform to a suitable field of view, and the illumination is automatically turned on to capture the first background image. After the background image is taken, the illumination is automatically switched off, and the specific photons emitted by the mouse are captured in the absence of an external light source (dark field). The superposition of the bright-field and dark-field background images can visually

display the location and intensity of the specific photons in the animal's body, completing the imaging operation. Images were taken at 0, 0.5, 1, 2, 4, 8, 24, 48 hours after gavage to observe the distribution of fluorescent microplastics in the mice. The colours on the images taken at the end of the study are the intensity of the fluorescent signals, and the more reddish the colour, the more the microplastics are accumulated.

S1.3. Metabolomics analysis data preprocessing process

The UHPLC-MS raw data were converted into the common format by Progenesis QI software (Waters, Milford, USA) through baseline filtering, peak identification, peak integral, retention time correction, and peak alignment. Then, the data matrix containing sample names, m/z, retention time and peak intensities was exported for further analyses. At the same time, the metabolites were identified by searching database, and the main databases were the HMDB (http://www.hmdb.ca/), Metlin (https://metlin.scripps.edu/) and the self-compiled Majorbio Database (MJDB) of Majorbio Biotechnology Co., Ltd. (Shanghai, China).

The data matrix obtained by searching database was uploaded to the Majorbio cloud platform (https://cloud.majorbio.com) for data analysis. Fistly, the data matrix was pre-processed, as follows: At least 80% of the metabolic features detected in any set of samples were retained. After filtering, the minimum value in the data matrix was selected to fill the missing value and each metabolic signature was normalized to the sum. To reduce the errors caused by sample preparation and instrument instability, the response intensities of the sample mass spectrometry peaks were normalized using the sum normalization method, to obtain the normalized data matrix. Meanwhile, the variables of QC samples with relative standard deviation (RSD) > 30% were excluded and log10 logarithmicized, to obtain the final data matrix for subsequent analysis.

S2. Supplementary results

S2.1. Body weight after drinking exposure to PS-NPs

The body weight of mice in all groups showed an increasing trend over time. Based on the observation showed that the body weight of mice in exposure group was higher than that of the control group, but the difference was insignificant ($p > 0.05$) (Figure S1).

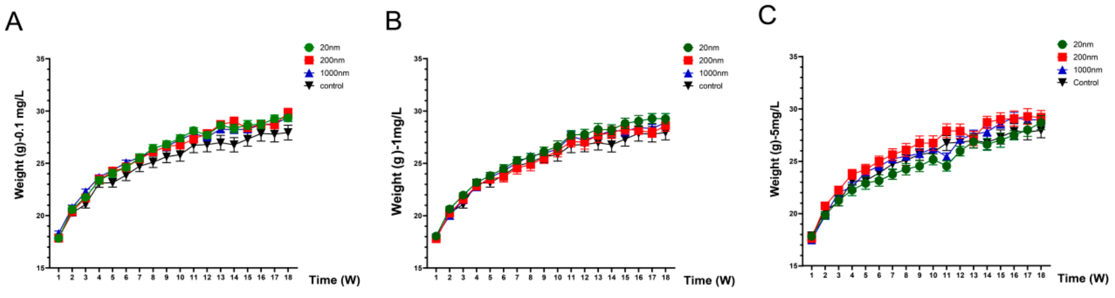

Figure S1: Body weight after drinking exposure to PS-NPs. Note: (**A**) 0.1 mg/L, (**B**)1 mg/L, (**C**) 5 mg/L.

S2.2. Changes in inflammatory factors and oxidative stress in testicular tissue after exposure

The results showed that after exposure to polystyrene nanoplastics, no changes in the levels of GSH-PX, TNF-α, and IL-1β were detected in the exposure groups when compared to the control group ($p > 0.05$) (Figure S2).

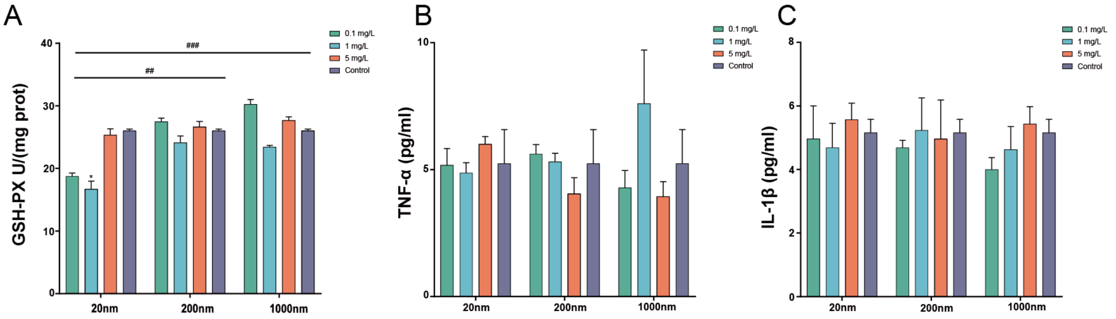

Figure S2: Changes in inflammatory factors and oxidative stress in testicular tissue after exposure. Note: (**A**) GSH-PX; (**B**) TNF-*α*； (**C**) IL-1β. "#" represents comparisons among exposure groups of different particle sizes, "##" is $p < 0.01$, "###" is $p < 0.001$.

S2.3. Analysis of the alpha diversity of gut microbiota

Analysis and comparison of the alpha diversity of gut microorganisms between the groups showed statistically significant differences in alpha diversity between the different particle size exposure groups, with the 20nm group having the highest alpha diversity and the 1000nm group the lowest (Figure S3).

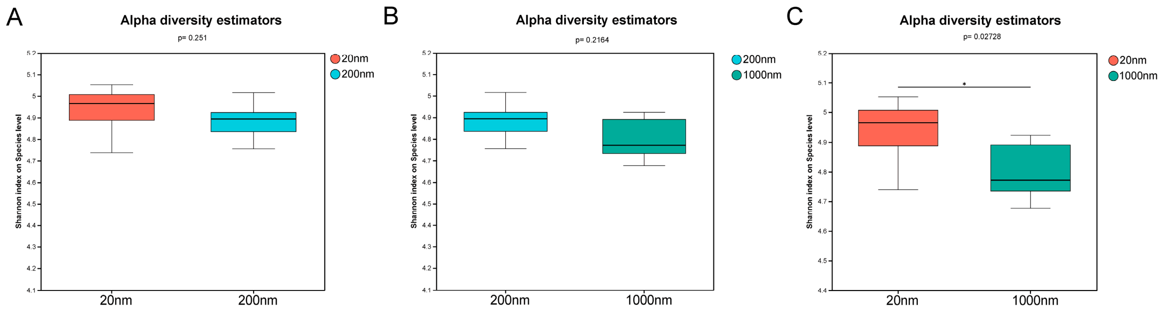

Figure S3: Analysis of the alpha diversity of gut microbiota. Note: (**A**) 20 nm vs 200 nm; (**B**) 200 nm vs 1000 nm; (**C**) 20 nm vs 1000 nm. "*" represents comparisons between exposure groups of different particle sizes and the control group. "*" is $p < 0.05$.

S2.4. LDA of Macrogenomic annotation

Linear discriminant analysis (LDA) revealed the microbial taxa that contributed significantly to the differences between groups, with the 20 nm exposure group contributing the most subgroup differences (Figure S4)

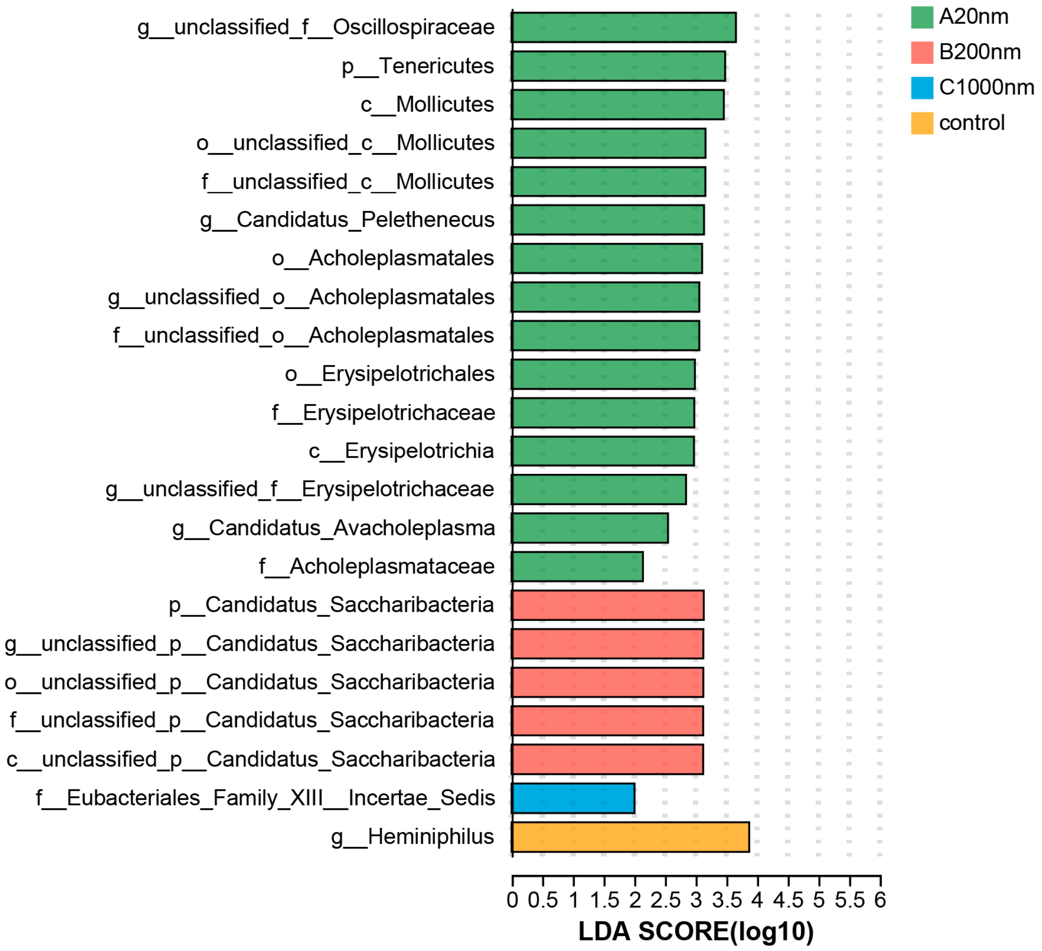

Figure S4: LDA of Macrogenomic annotation. Note: The LDA discriminant bar chart counts the microbial taxa with significant effects among two or more groups, and the LDA scores obtained from the LDA analysis, the larger the LDA scores, the greater the impact of species abundance on the differential effects.

S2.5. Imaging small animals in vivo

PS-NPs were found to enter the gastrointestinal tract section of the mice after 0.5 h of gavage and transferred to other parts of the body as time progressed (see Figure S5).

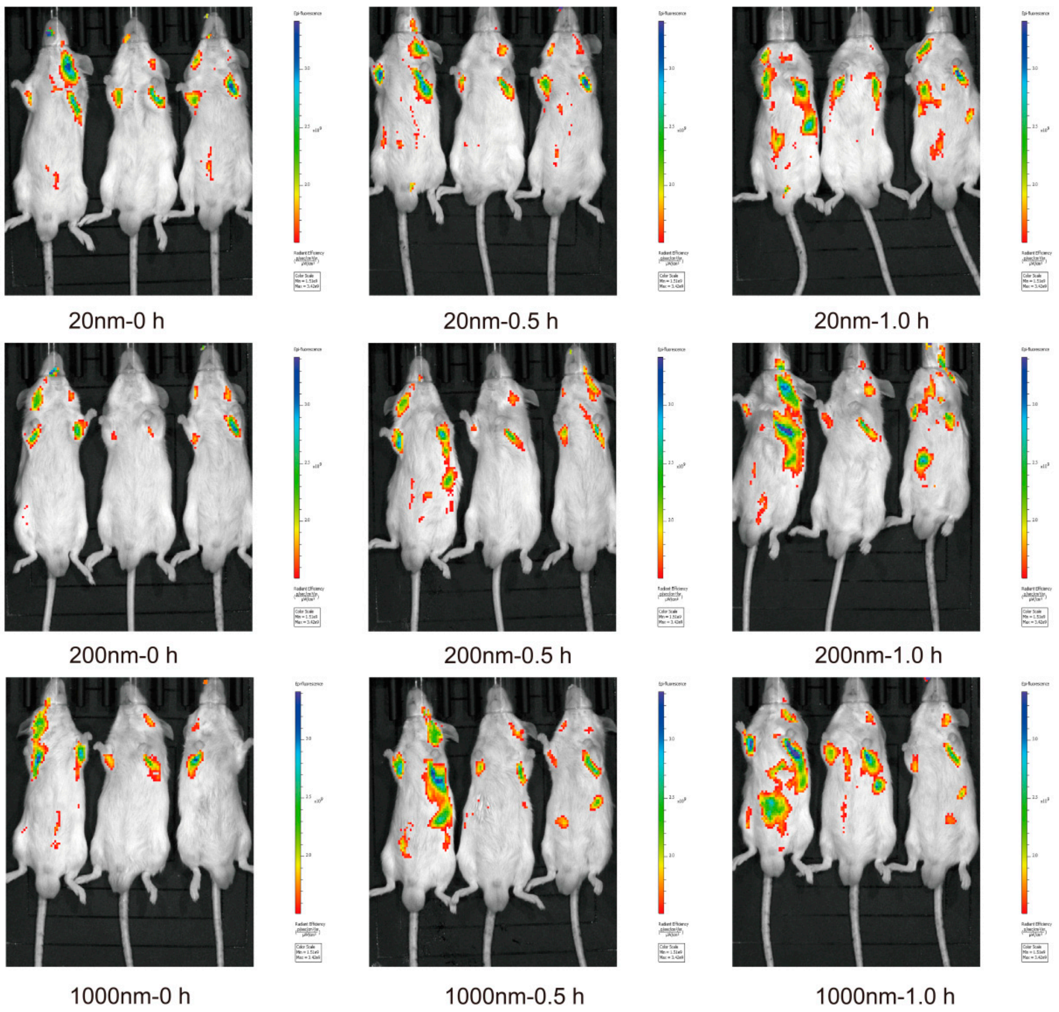

Figure S5: Imaging small animals in vivo.
